# The Effect of Daily Sedation-Weaning Application on Morbidity and Mortality in Intensive Care Unit Patients

**DOI:** 10.7759/cureus.2062

**Published:** 2018-01-13

**Authors:** Selcuk Kayir, Hulya Ulusoy, Guvenc Dogan

**Affiliations:** 1 Anesthesiology and Reanimation, Hitit University Erol Olcok Training and Research Hospital; 2 Anesthesiology and Reanimation, Karadeniz Technical University

**Keywords:** intensive care, sedation, weaning, daily awakening

## Abstract

Background/aims

Sedation is one of the most important components of intensive care unit (ICU) in patients who are mechanically ventilated at intensive care conditions. As a result of sedation and analgesia in the intensive care unit, the patient is to be awakened a comfortable and easy process. The aim of the study is to demonstrate the effects of day-time sedation interruptions in intensive care patients.

Material and methods

We made a retrospective review of 100 patients who were monitored, mechanically ventilated and treated at our intensive care unit between January 2008 and January 2013. Patients were divided into two groups, including Group P (continuous infusion of sedative agent) and Group D (daily sedation interruptions - daily recovery).

Demographics, mechanical ventilation time, stay at intensive care unit, hospitalization period, time of first weaning, success of weaning, ventilator-related pneumonia (VRP), total doses of drugs, re-intubation frequency, Acute Physiology and Chronic Health Evaluation II (APACHE II), Sequential Organ Failure Assessment (SOFA) scores and mortality rates of patients were compared. Ramsay Sedation Score (RSS) was used to evaluate the level of sedation. Considering that ideal sedation level is "3" with RSS, RSS < 3 is considered as mild sedation, while RSS > 3 is considered as deep sedation.

Results

There was no difference between demographics of patients. Mechanical ventilation period was significantly longer in Group P than Group D (p < 0.001). When stay at ICU unit was considered, ICU stay was significantly longer in Group P than Group D (p < 0.001). No statistically significant difference was found between two groups with respect to hospitalization period. In inter-group comparison, time to start first weaning was significantly late in Group P than Group D (p < 0.05). There was no difference between groups in terms of frequency of success of weaning and mortality rate (p > 0.05). In inter-group comparison the frequency of reintubation viewed in Group D was significantly less than in Group P (p < 0.05). Considering development of VRP, it was significantly more common in Group P in comparison with Group D (p < 0.05). No statistically significant difference was found between groups in terms of doses of sedative agents (p > 0.05). Considering doses of opioid analgesics, the total dose of fentanyl was significantly higher in Group P than Group D (p = 0.04), while no difference was found for doses of morphine (p > 0.05). Again, no statistical difference was found in doses of muscle relaxant agents (p > 0.05).

Conclusion

It was observed that the sedation technique with daily interruption is superior to continuous infusion of sedatives. Accordingly, we believe that daily weaning will make positive contributions to patients who are mechanically ventilated at intensive care unit.

## Introduction

To increase compliance with treatment and to reduce anxiety and pain, sedative and analgesics are used for patients linked to mechanical ventilators (MV) in intensive care units (ICU) [1]. Monitoring of mild sedation is necessary. Insufficient or excessive sedation may cause harmful effects in the patient. Insufficient sedation may be displayed as symptoms like hypertension, tachycardia, discomfort, hypoxia, hypercapnia and struggling with the ventilator. Excessive sedation may cause unwanted situations such as hypotension, bradycardia, coma, respiratory depression, ileus, renal failure, venous stasis and immunosuppression [2,3]. Lengthening of the sedation duration is proposed as a risk factor for the development of ventilator-related pneumonia (VRP). Delays in patient healing lengthen the duration of mechanical ventilation and stay in the intensive care unit and hospital increasing hospital costs [4,5].

Ideal administration of sedative or analgesic medication begins with a low dose and increases the dose according to requirements. To reduce the risk of overdose, there are benefits to assessing the medication administration and consciousness situation at regular intervals. Additionally, it is recommended to evaluate consciousness situations at least once in every 24-hour interval [6].

Pain causes tachycardia, increases in the use of oxygen in the myocardium, hypercoagulability and increased catabolism in intensive care patients. This situation clinically may cause intense anxiety, agitation and other medical problems in the patient. For pain treatment in intensive care primarily narcotic agents are used. However, the half-life of commonly used opioid analgesics is long and doses that provide required analgesia may cause severe side effects (histamine discharge, hypotension, respiratory depression, gastrointestinal side effects). While low doses of opioids provide analgesia, they do not cause anxiolysis; however high doses may have sedative effects [7].

Two methods are used to administer sedation to patients given mechanical ventilation treatment in the intensive care unit. The first and most common method is to cease sedation during the day (daily sedation cuts) and administer sedative medication according to the clinical tableau and sedation scores after patient examination [5]. The other method is to use sedation scores from a sedation protocol prepared by the doctor with nurse administered sedation.

The aim of this study is to investigate the effects of daily sedation cessation procedure on the mechanical ventilator duration, stay in intensive care, stay in the hospital, spontaneous respiratory attempt (SRA) success, morbidity and mortality for intubated patients linked to mechanical ventilator in intensive care. According to the results of the obtained data, the reliability and feasibility of daily sedation cessation for patients is determined.

## Materials and methods

This study was clinical research with retrospective design completed in the Anesthesiology and Reanimation Department of Karadeniz Technical University Faculty of Medicine. After receiving permission from Karadeniz Technical University Clinical Research Ethics Committee (2013/15), data from patients monitored and treated in the Anesthesiology and Reanimation Department Intensive Care Unit from January 2008 to January 2013 were investigated.

In the study, 50 patients abiding by the following criteria were included in the study. Patients were divided into two groups as Group P (sedation protocol) and Group D (Daily awakening).

Inclusion criteria for the study

·         Adult patients (18 years and older) on mechanical ventilation for two days and longer

Exclusion criteria for the study

·         Not on mechanical ventilation

·         Extubated within the first two days of MV

·         Cardiac arrest developed

·         Death before extubation

·         Age below 18 years

·         Requiring MV at home on discharge from ICU

·         Tracheostomy performed

·         Intracranial event

·         Muscle relaxant agents used for more than 24 hours

·         Sedative infusion for more than 24 hours at external center before intensive care.

Patients, abiding by the above criteria, given sedation-analgesia infusion according to conventional sedation protocol in the intensive care unit were included in Group P. Patients given infusion according to sedation protocol but with a break in sedative infusion each morning (daily awakening) were included in Group D. According to routine administration in our clinic, both groups had sedation degree evaluated according to the Ramsay Sedation Score (RSS). Medication doses were titrated according to the patient’s RSS. As a result of evaluations, if patients were agitated (RSS = 1) additional intravenous bolus injections of sedative and opioids medications were given and infusion dose increased. If patients had RSS > 3 infusion dose was reduced and if RSS = 3 sedative-opioid dose was continued at the same dose.

**Figure 1:**
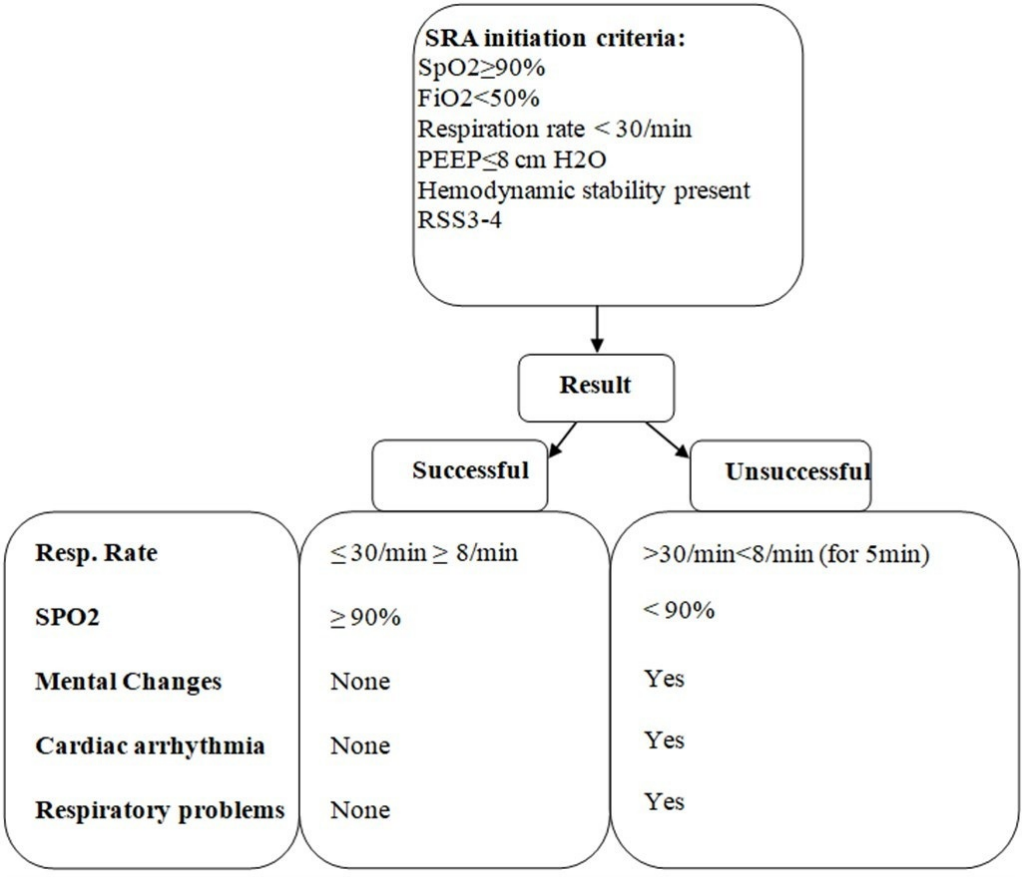
Scheme for spontaneous respiration attempt. RSS: Ramsay Sedation Score; SRA: Spontaneous respiratory attempt.

Note: Patients who were not extubated within seven days from beginning of first spontaneous respiratory attempts were accepted as being unsuccessful in disconnecting from ventilator.

Similarly, according to routine administration at our clinic, SRA was begun for both groups of patients based on the following table (Figure 1).

Data for all patients included in the study were investigated until mortality occurred or the patient was discharged from the intensive care unit. The following data were recorded from the patients’ files:

•          Demographic data; gender, age, reason for admittance, admission Acute Physiology and Chronic Health Evaluation II (APACHE II) and Sequential Organ Failure Assessment (SOFA) scores

•          Admission diagnosis and date to the intensive care unit

•          Duration of mechanical ventilator support (day)

•          Duration of stay in intensive care (day)

•          Duration of stay in hospital (day)

•          First weaning time

•          Weaning success

•          Reintubation incidence

•          Development of VRP or not

•          Total sedative agent dose consumed

•          Total additional sedative agent dose consumed

•          Total bolus neuromuscular blocker agent consumed

•          APACHE II and SOFA scores on admission (0 day) and on the 4th, 7th and 14th day of treatment

•          Development of mortality or not

Statistical Evaluation

Statistical analysis of data used the “Statistical Package for Social Sciences” (SPSS) for Windows Release 13.0.1 program (license no: 9069727).

Normal distribution of data obtained from measurements was investigated with the Kolmogorov–Smirnov test. Comparison of data used the Student's t-test for those with normal distribution and the Mann-Whitney U test for those with non-normal distribution.

Comparison of qualitative data used the chi-square test. Comparison of repeated measurements within the groups used variance analysis for repeated measurements (post hoc paired t test).

Data obtained for measurements are given as arithmetic mean ± standard deviation, with numbers of organs in failure given as median, counted data are given as %. Significance level was taken as p < 0.05.

## Results

**Figure 2:**
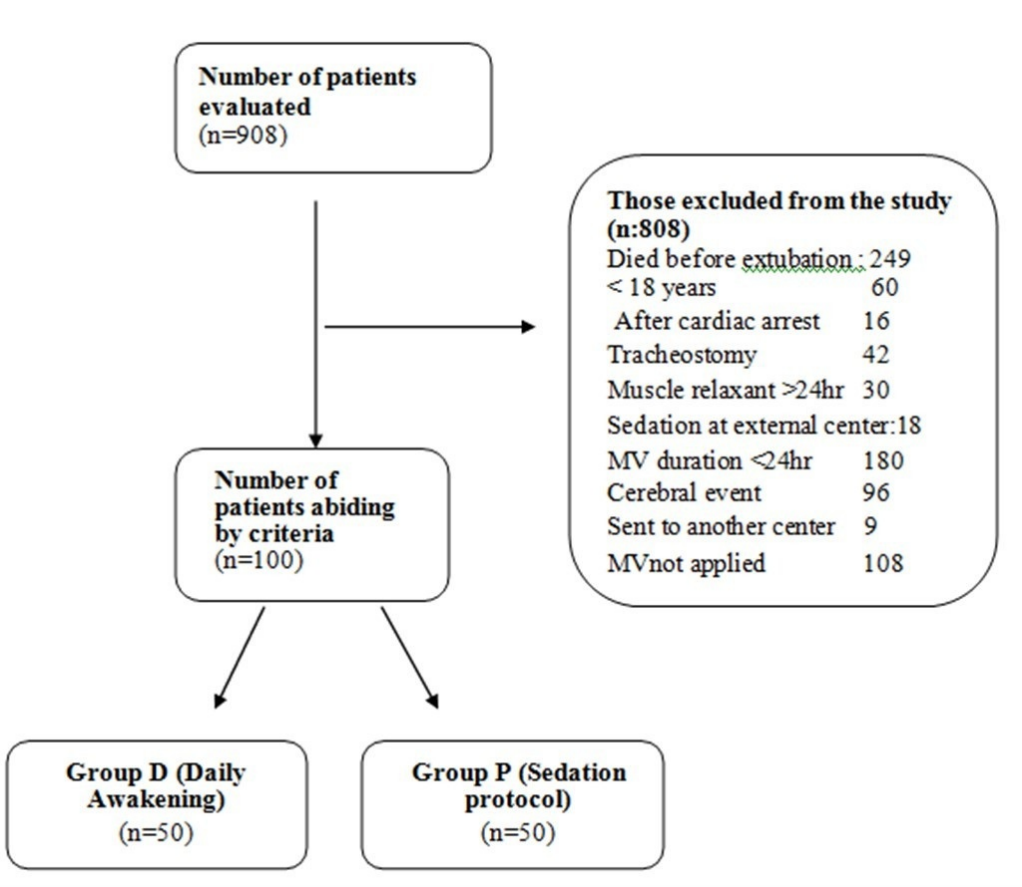
Patient randomization scheme.

This study included patients treated in the anesthesiology and reanimation intensive care unit of Karadeniz Technical University from January 2008 to January 2013 who abided by the study criteria (Figure 2).

A total of 100 patients abiding by the criteria included 41 (41%) females and 59 (59%) males, with mean age 47.2 ± 20.19 (mean ± SD). Trauma was the most common cause for stay in intensive care, with other reasons given in the table below. Generally, 13 patients required reintubation and 24 patients developed VRP. Ten of the patients included in the study were exitus. Apart from APACHE II score, there was no significant difference identified between the groups in terms of demographic data (p > 0.05) (Table 1).

**Table 1:** Distribution according to demographic characteristics. Data are given as mean ± standard deviation * : Statistically significance between groups (p < 0.05). APACHE II: Acute Physiology and Chronic Health Evaluation II; ICU: Intensive care unit; SOFA: Sequential Organ Failure Assessment.

	Group P (n = 50)	Group D (n = 50)	p-value
Age	48.52 ± 20.72	45.88 ± 19.76	0.516
Gender			
--Male	30	29	0.977
--Female	20	21	0.971
Intervention			
--APACHE II	17.16 ± 3.99	14.76 ± 4.19	0.004*
--SOFA	5.9 ± 3.53	5.44 ± 2.86	0.393
Reason for ICU stay			>0.05
--Trauma	22	17	
--Postoperative	11	16	
--Sepsis	2	1	
--Intoxication	4	1	
--Respiratory failure	11	15	

**Figure 3:**
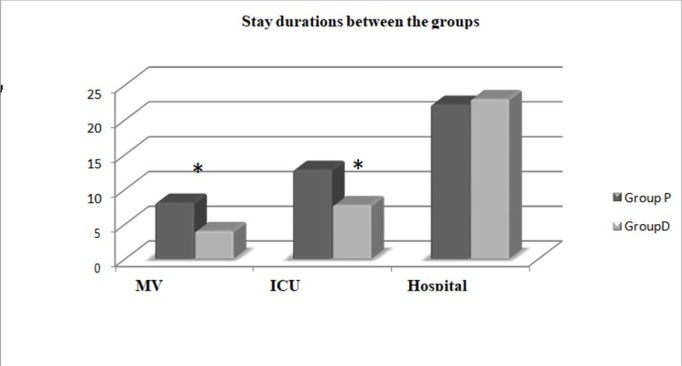
Mechanical ventilator, intensive care and hospital stay durations between the groups. *:Statistically significant difference between groups (p < 0.001). ICU: Intensive care unit; MV: Mechanical ventilator.

When the MV durations are compared between the two groups, it was significantly shorter in Group D compared to Group P (p < 0.001). When the ICU stay duration is compared between the two groups, it was again statistically significantly shorter in Group D compared to Group P (p < 0.001). There was no statistically significant difference between the hospital stay duration in both groups (p > 0.05) (Table 2) (Figure 3).

**Table 2:** Mechanical ventilator, intensive care and hospital stay durations of the group. * : Statistically significance between groups (p < 0.05). ICU: Intensive care unit; MV: Mechanical ventilator.

	Group P (n = 50)	Group D (n = 50)	p-value
MV use duration (day)	8.1 ± 4.9	4.02 ± 3.71	<0.001*
ICU stay duration (day)	12.82 ± 6.08	7.74 ± 4.5	<0.001*
Hospital stay duration (day)	22.18 ± 12.84	22.96 ± 21.68	0.183

**Figure 4:**
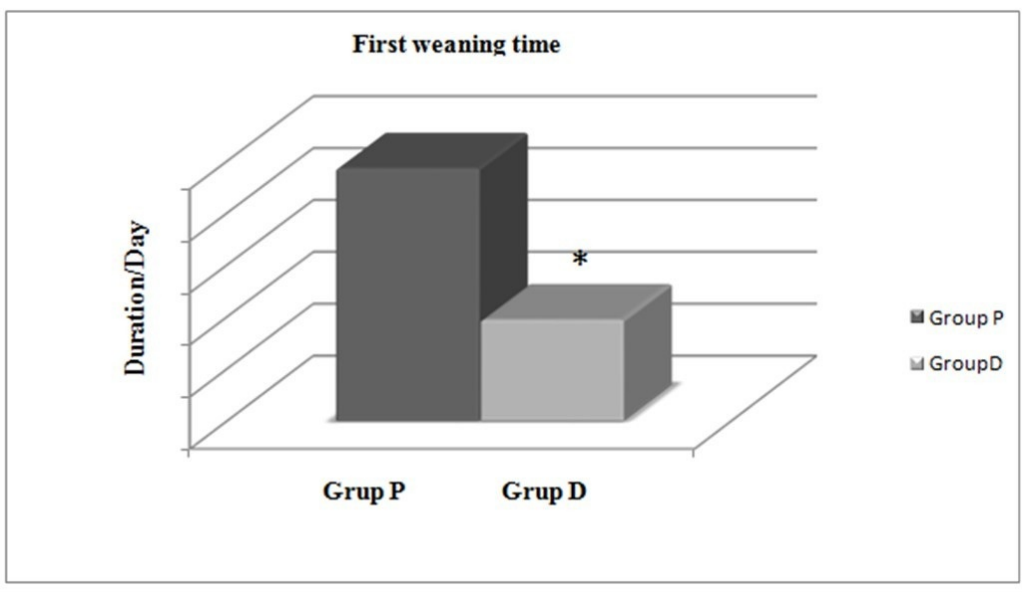
Comparison of first weaning time between groups. *: Statistically significant difference between the groups (p < 0.05).

When first weaning times are examined between the groups, the first weaning attempt for Group P was 4.86 ± 3.98 days, while it was 1.94 ± 2.64 days in Group D. This comparison between the groups found that the first weaning initiation in Group D was statistically significantly earlier than Group P (p < 0.05) (Figure 4).

**Figure 5:**
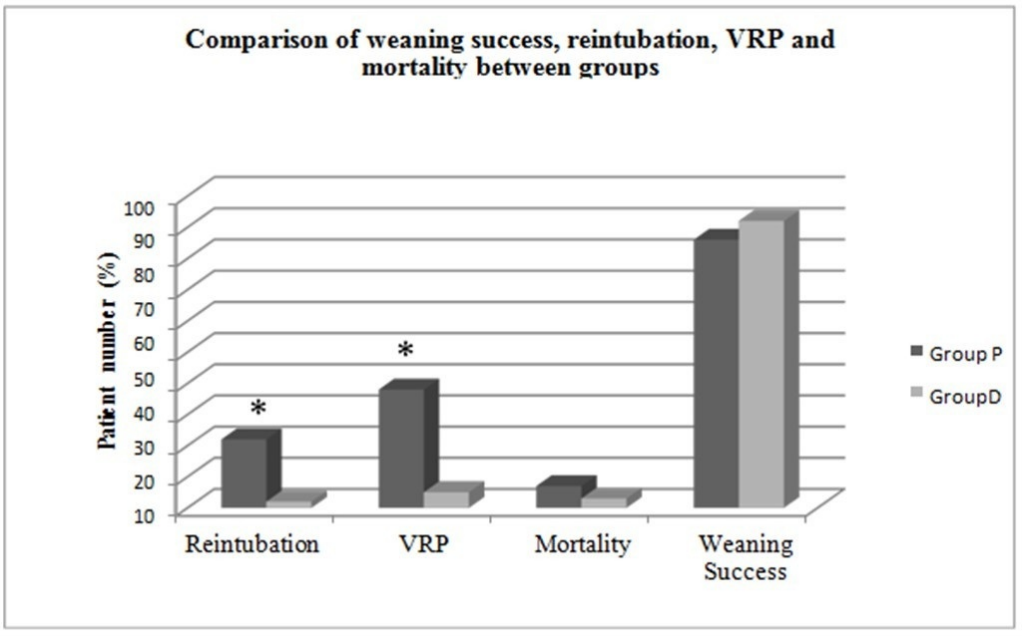
Comparison of weaning success, reintubation, VRP and mortality between groups. *= Statistical significance between groups (p < 0.05)​​​​​ VRP: Ventilator-related pneumonia.

There was no statistically significant difference between the groups in terms of weaning success (p = 0.523). Reintubation was performed less often in Group D by a statistical difference compared to Group P (p < 0.05). When VRP development was compared, it was observed less in Group D by a statistically significant amount compared to Group P (p < 0.05). When mortality rates are compared between the two groups, there was no statistically significant difference found (p = 0.317) (Table 3) (Figure 5).

**Table 3:** Comparison of weaning success, reintubation, VRP and mortality between groups. * = Statistical significance between groups (p < 0.05). VRP: Ventilator-related pneumonia.

Parameters	Group P (n = 50)	Group D (n = 50)	p-value
Weaning success	43 (86%)	46 (92%)	0.523
Reintubation	11 (22%)	2 (4%)	0.017*
VRP	19 (38%)	5 (10%)	0.002*
Mortality	7 (14%)	3 (6%)	0.317

The APACHE II and SOFA scores in both groups on day first and the fourth, seventh and 14th days are shown in Table 4.

**Table 4:** Comparison of APACHE II and SOFA scores between both groups. * = Statistical significance between groups (p < 0.05) APACHE II: Acute Physiology and Chronic Health Evaluation II; SOFA: Sequential Organ Failure Assessment.

	Group P (n = 50)	Group D (n = 50)	p-value
APACHE II			
0 day	17.16 ± 3.99	14.76 ± 4.19	0.004*
4^th^ day	15.02 ± 4.48	9.14 ± 4.19	<0.001*
7^th^ day	13.61 ± 6.17	9.00 ± 3.64	0.002*
14^th^ day	9.41 ± 3.53	11.33 ± 4.93	0.420
SOFA			
0 day	5.9 ± 2.47	5.44 ± 2.86	0.393
4^th^ day	5.12 ± 2.95	1.92 ± 2.14	<0.001*
7^th^ day	4.36 ± 4.21	1.52 ± 2.41	<0.001*
14^th^ day	1.24 ± 1.09	3.00 ± 4.35	0.866

In the table above, there was a statistically significant difference between the APACHE II scores in the two groups on day first, and the fourth and seventh days and between SOFA scores in the two groups on the fourth and seventh days (p < 0.05) (Table 4).

**Figure 6:**
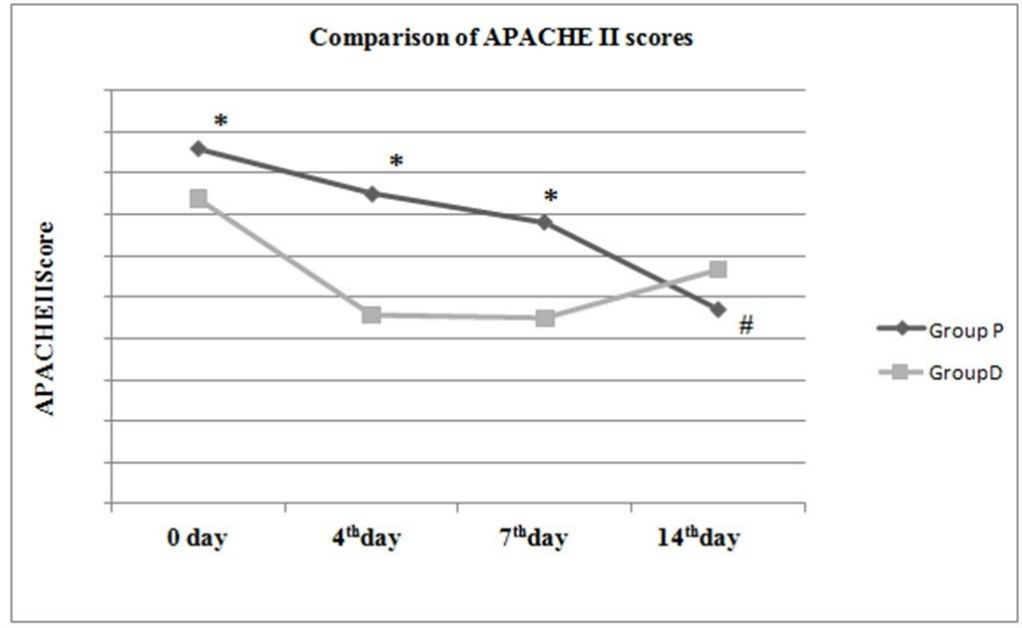
Comparison of APACHE II scores between and within groups. ​​​​​* = Statistical significance between groups (p < 0.05) # = Statistical significance within group compared to 0 day (p < 0.05) APACHE II: Acute Physiology and Chronic Health Evaluation II.

**Figure 7:**
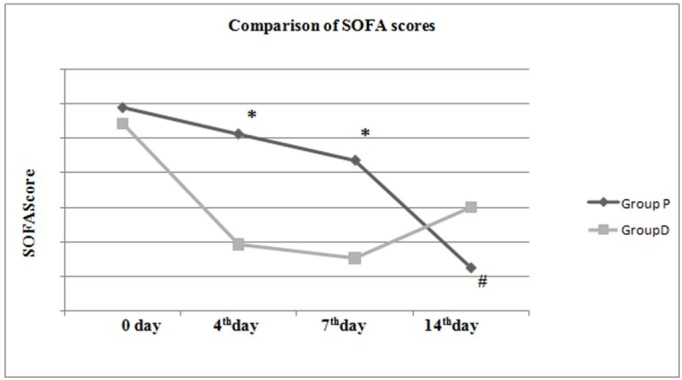
Comparison of SOFA scores between and within groups. * = Statistical significance between groups (p < 0.05) # = Statistical significance within group compared to 0 day (p < 0.05) SOFA: Sequential Organ Failure Assessment.

Evaluation within the groups found that in Group P there was no difference in the APACHE II scores on the fourth and seventh days compared to day 0 (p > 0.05), while the score on the 14th day was found to be significantly low (p < 0.05). In Group P there was no difference between SOFA scores on the fourth and seventh days compared to day first (p > 0.05), while the 14th day score was significantly low (p < 0.05). Evaluation within the group found that in Group D there was no significant difference between the APACHE II and SOFA scores (p > 0.05) (Figures 6-7).

The total sedative, opioid analgesic and muscle relaxant consumption in both groups of patients were compared. In our intensive care unit midazolam, propofol, thiopental and dexmedetomidine are used as sedative agents, fentanyl and morphine are used as opioids analgesics and cisatracurium is used as muscle relaxant.

**Figure 8:**
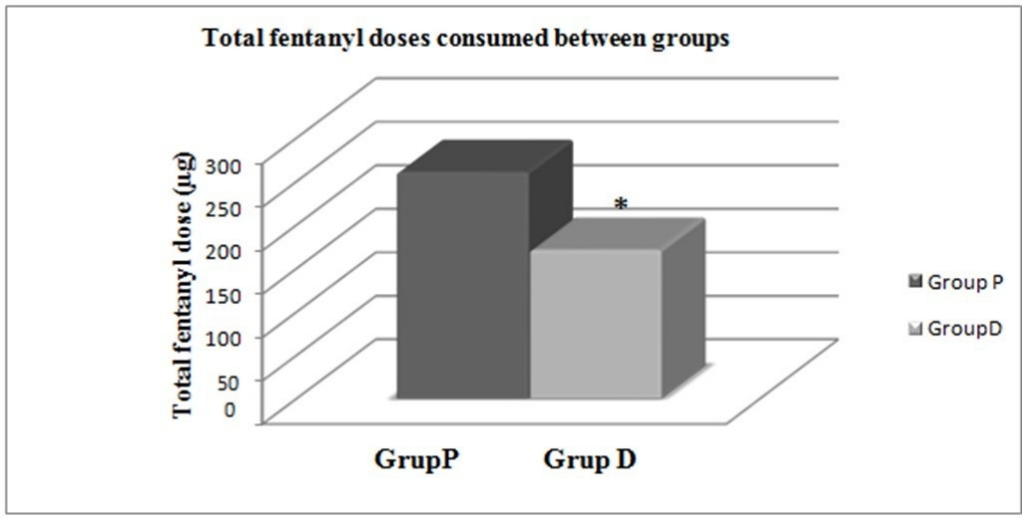
Comparison of total fentanyl doses consumed between groups. * : Statistical significance between groups (p < 0.05).

There was no statistically significant difference between the total sedative medication doses consumed between the groups (p > 0.05). While the total fentanyl dose consumed by patients among opioid analgesia doses was significantly low in Group D compared to Group P (p = 0.04), there was no significance identified in terms of total consumed morphine dose (p > 0.05). There was no statistical significance identified in terms of total consumed muscle relaxant doses (p > 0.05) (Table 5) (Figure 8).

**Table 5:** Comparison of total medication consumption of patients. * : Statistical significance between groups (p < 0.05)

	Group P (n = 50)	Group D (n = 50)	p-value
Sedative agents			
Midazolam (mg)	30.18 ± 26.05 (n = 46)	18.92 ± 12.3 (n = 38)	0.11
Propofol (mg)	330.5 ± 211.49 (n = 8)	343.26 ± 221.26 (n = 5)	1
Thiopental (mg)	771.23 ± 771.83 (n = 3)	740.66 ± 490.03 (n = 3)	0.827
Dexmedetomidine (µg)	167.41 ± 249.08 (n = 5)	109.90 ± 86.49 (n = 4)	0.462
Opioid agents			
Fentanyl (µg)	260.64 ± 152.79 (n = 36)	171.19 ± 83.97 (n = 31)	0.04*
Morphine (mg)	25.02 ± 17.15 (n = 14)	32.06 ± 15.75 (n = 9)	0.457
Muscle relaxant			
Cisatracurium (mg)	1.34 ± 1.21 (n = 11)	2.13 ± 1.51 (n = 8)	0.227

## Discussion

Nearly 1/3 of patients in intensive care require mechanical ventilation and commonly require analgesia and sedation. In spite of clear benefits clinically, medications used for sedation have significant side effects making medication and dose choice difficult for clinicians [8].

Though there are many indications for sedation and analgesia in intensive care, reducing pain takes most important place. Patients with mechanical ventilation commonly have underlying diseases and pain due to routine procedures in intensive care, with pain having significant side effects like increased endogenous catecholamine activity, anxiety and delirium [9]. It is known that these patients may experience post-traumatic stress disorder due to bad experiences in intensive care. Patients using muscle relaxants especially have increased sedation requirements [10].

Continuous infusion of elderly patients or patients with liver failure has increased the risk of over-sedation [11]. As much as medication choice, the method of administering medication, in other words, sedation method, affects the duration of mechanical ventilation and ICU stay. Medications may be administered as both interval bolus dose and continuous infusion. To prevent agitation and ensure adaptation to intensive care conditions, many intensive care patients require aggressive sedation and commonly continuous sedative infusion is chosen [12]. However, in recent years, daily breaks to sedative infusion or sedation based on a determined protocol have been shown to be more advantageous compared to sedation methods administering continuous sedatives [5,12,13]. Continuous sedation is known to increase the mechanical ventilation duration, stay in intensive care and hospital, organ failure and reintubation rates.

APACHE II and SOFA score are scoring systems used to monitor treatment and estimate prognosis of intensive care patients. From the start of treatment a fall in scores indicates treatment is advancing positively [14]. In our clinic, monitoring of patients in the ICU uses the APACHE II and SOFA scores. In our study the APACHE II scores on the 0 day, 4th and 7th days were observed to be significantly low in the group with sedation breaks. SOFA score was observed to be significantly low on the 4th and 7th days in the group with sedation breaks. Though these results were statistically significant, the difference in the scores was low and was not observed to cause clinical change.

A randomized-blind study by Strom, et al. showed that duration on mechanical ventilation, intensive care and hospital stay were better in the group not using sedation [15]. Studies investigating the correlation between continuous sedation and ventilator-related pneumonia found it was an independent risk factor [16]. In our study, the VRP incidence in the sedation group with daily breaks was observed to be lower by a significant level compared to the continuous sedative infusion group. The reason for this is that patients with daily sedation breaks stayed on mechanical ventilation for shorter duration and we believe VRP development was lower linked to this.

Wunsch, et al. in a retrospective study of 171 ICU in the United States of America found that 51.5% of 109,671 patients on mechanical ventilation received one or more sedative infusions, with sedative use 39.7% in 2001 rising to 66.7% in 2007. Of those receiving sedation, 81% were given propofol, 31% benzodiazepine, and 34% dexmedetomidine with patients given mechanical ventilation for more than 96 hours using more propofol [17]. Salluh, et al. in a study of 1015 patients investigated the sedation and delirium administration of intensive care doctors in Brazil and reported that 88.3% of participants applied a sedation scoring system, while 62.8% did not mention sedation targets and 68.3% did not mention daily sedation breaks. More than half of participants applied a sedation protocol, with midazolam (97.8%), fentanyl (91.5%) and propofol (55%) commonly used as sedative [18]. In our intensive care, unless there are contraindications, mainly benzodiazepine infusion is used and fentanyl infusion as analgesic is chosen. According to the patient’s clinical situation and sedation scales, if the sedative and analgesic agents are insufficient for the target score, infusion dose is increased with bolus dose; if necessary, the transition to another agent from the same group is made.

Reschreiter, et al. in a study of intensive care units in England found that 88% used a sedation scoring system, with the Ramsay sedation score commonly used (66.4%) with 80% applying sedation guidelines and 78% stating they applied a break in sedation during the day. For short-term sedation, propofol and alfentanil were commonly used, with propofol, midazolam, and morphine chosen for longer periods [19]. We found that in our practice, the total fentanyl dose consumed by patients among opioid analgesia doses was significantly low in Group D compared to Group P. Together with similarities and different applications observed between countries, there is a need for multi-center studies to identify the current administration in our country and then to increase quality and standardization.

A retrospective study of 128 patients by Schweickert, et al. identified that for patients with mechanical ventilation, when sedation is administered with a daily break, the mechanical ventilation and intensive care stay durations significantly shortened, and there was a reduction in complications. A daily break in sedative infusion prevents accumulation of sedative medication, allows titration of medication doses and thus ideal sedation levels are more easily reached and mild sedation levels are more common. As the sedative medication accumulation is less in the group with daily break, these patients make efforts at spontaneous respiration in a shorter period compared to patients with continuous sedation infusion. The result is that if there is no underlying obstacle, it was concluded that these patients have earlier weaning initiation compared to other groups [20]. In our study, the group with daily sedation breaks had first weaning initiation significantly earlier compared to the continuous sedation infusion group.

The greatest disadvantage of a daily sedation break is patients may wake to unwanted agitation. Agitated patients are observed to have more common complications like incompliance with mechanical ventilation, and removal of venous lines, nasogastric tube or endotracheal tube by patients [21]. In our study complications like self-extubation incidence and linked reintubation were determined to be less common in the group given daily breaks in sedative infusion. We believe the reason for this is that agitation of patients was quickly noticed by the intensive care team and sedation levels were rapidly titrated. Thus, the desired sedation levels in patients were obtained using additional medication.

A double-blind, randomized, multicenter study by Mehta, et al. compared a patient group with daily sedation breaks and a patient group with sedation protocol. While there was no difference between the groups in terms of hospital stay, MV duration, ICU stay and mortality, the total midazolam infusion dose was significantly lower in the group with daily sedation breaks. There was no difference observed in additional sedative and opioids doses [22]. In our study, the fentanyl dose was significantly lower in the daily awakening group, and though there was no difference in the midazolam infusion, it appeared lower doses were used. Daily breaks in sedative infusion lessen the sedation level and cause shortening of both sedation duration and mechanical ventilator duration. Again the Canada ICU survey study by Mehta, et al. determined only 29% of patients had sedation/analgesia protocol used and of these, only 40% reported they used daily breaks. In the United States of America it was determined 64% of patients had sedation/analgesia protocol applied and of these, only 40% reported they used daily sedation breaks. Among the causes of low use of sedation breaks were low number of nurses for daily breaks, concerns about stopping sedative agents and patient discomfort.

Brattebo, et al. researched the effects of scoring systems and sedation protocols in patients receiving mechanical ventilator support in the surgical ICU. They identified that use of sedation protocol and motor activity assessment scales shortened the mechanical ventilation and ICU stay durations. They reported the reason for this was that the patient group with sedation protocol and scoring system used had less medication accumulation and as a result these types of patients had earlier recovery [23]. In our intensive care unit, we use the Ramsay sedation score to determine sedation levels for patients. We believe this is helpful to prevent medication accumulation in patients. A randomized controlled study by Anifantaki, et al. assessed 97 patients linked to mechanical ventilation in the ICU. They compared patients with daily sedation breaks and continuous infusion. In both groups, the mechanical ventilation duration, hospital stay, ICU stay, and mortality were evaluated and were observed to be similar. In both groups again the total sedation, analgesia and muscle relaxant doses consumed were similar. They stated that the conclusion was that daily sedation breaks had no negative effects on patients, with the advantage that patients showed earlier signs of awakening [24].

A prospective observational study by Kollef, et al. compared patients with interval bolus sedation and continuous sedative infusion and identified that those with continuous sedation had longer mechanical ventilation, intensive care and hospital stay durations, with increased rates of organ failure and reintubation. Though the researchers arranged sedative infusion dose according to patient age and disease severity, they proposed mechanical ventilation and intensive care stay durations increased and recommended research into the use of sedation protocols [12].

A study by Kress, et al. of intensive care patients compared the long-term psychological effects on patients with daily sedation breaks with those given continuous sedation infusion. While making this comparison, they used scales showing the patients’ psychological and physiological situations. They found that daily sedation breaks did not cause negative psychological results in intensive care patients. Additionally, the post-traumatic stress disorder rates were lower in the patient group with sedation breaks. This was related to less observation of symptoms linked to post-traumatic stress disorder in the group with daily breaks. Again the same study identified that daily breaks in sedation prevented deep sedation, shortened mechanical ventilation, and intensive care stay durations and reduced the sedative doses used [25]. A randomized, controlled study of 336 patients by Timothy, et al. compared a daily awakening group with continuous sedation group. Patients in both groups had spontaneous respiration attempts. The result was that for patients with daily awakening, the ICU stay duration and hospital stay duration were significantly lower. Though there was no difference in the 28-day mortality, it was observed that the 1-year mortality was lower in the daily awakening group. The sedation/analgesia doses used were similar in both groups. The self-extubation rate in the daily awakening group was higher, though the reintubation rate after self-extubation was similar in both groups [26]. In our study, the reintubation rates were observed to be higher in the group with continuous sedation.

A study by Yılmaz, et al. compared the effect on mechanical ventilation duration of sedation administration with nurse-controlled sedation based on a defined protocol prepared by doctors with a doctor-controlled sedation with daily breaks. In both groups, the ICU stay duration and mortality rates were similar, while the sedation and MV duration in the daily break group were found to be significantly shorter [27]. A study by O’Connor, et al. reviewed eight studies (three randomized controlled, two cohort, three descriptive) from 1995 to 2006 and reported that interval sedation reduced ventilation duration, intensive care unit stay, complications and post-traumatic stress syndrome [28]. Our study results observed that patients with daily sedation breaks had significantly shorter MV duration and ICU stay duration. A randomized controlled study by Kress, et al. assessed 128 patients linked to mechanical ventilator. Patient groups with continuous sedation and sedation administration with daily breaks during mechanical ventilation treatment were compared and daily breaks in sedation prevented deep sedation, shortened the mechanical ventilation and intensive care stay durations and reduced the sedative medication and analgesia doses used [5].

A double-blind randomized controlled study by Weisbrodt, et al. compared the reliability and feasibility of a sedation protocol with daily breaks for patients linked to MV. In both groups examined MV duration, ICU and hospital stay durations and mortality rates were similar. Though the sedation and opioid doses used did not reach significance, there were lower doses of medications consumed in the daily break use group. They stated that daily breaks were reliable and feasible [29].

## Conclusions

We investigated the sedation techniques of patients linked to a mechanical ventilator at the anesthesia and reanimation intensive care unit at our clinic. In our study, Group P included patients given continuous sedative infusion according to a sedation protocol, while Group D included patients given sedative infusion with daily sedation breaks (daily awakening). In the group with daily sedation breaks we observed:

1) Shorter duration on mechanical ventilator

2) Lower incidence of ventilator-related pneumonia

3) Shorter stay in the intensive care unit

4) Lower total opioids dose consumed

5) Earlier time for first weaning initiation

6) Lower APACHE II and SOFA scores

In conclusion, the group with daily sedation breaks appeared superior to the group with continuous sedation. As a result, we believe that giving daily sedation breaks to patients in intensive care linked to mechanical ventilator should be the sedation technique chosen.

## Appendices

In our study, we were convinced that daily awakening had a positive effect on patients.
